# A comprehensively prognostic and immunological analysis of actin-related protein 2/3 complex subunit 5 in pan-cancer and identification in hepatocellular carcinoma

**DOI:** 10.3389/fimmu.2022.944898

**Published:** 2022-09-06

**Authors:** Shenglan Huang, Liying Sun, Ping Hou, Kan Liu, Jianbing Wu

**Affiliations:** ^1^ Department of Oncology, The Second Affiliated Hospital of Nanchang University, Nanchang, China; ^2^ Jiangxi Key Laboratory of Clinical and Translational Cancer Research, The Second Affiliated Hospital of Nanchang University, Nanchang, China; ^3^ Department of Hepatobiliary Surgery, The Second Affiliated Hospital of Nanchang University, Nanchang, China

**Keywords:** ARPC5, prognosis, biomarker, immune, pan-cancer, hepatocellular carcinoma

## Abstract

**Background:**

Actin-related protein 2/3 complex subunit 5 (ARPC5) is one of the members of actin-related protein 2/3 complex and plays an important role in cell migration and invasion. However, little is known about the expression pattern, prognosis value, and biological function of ARPC5 in pan-cancer. Thus, we focus on ARPC5 as cut point to explore a novel prognostic and immunological biomarker for cancers.

**Methods:**

The public databases, including TCGA, GTEx, and UCEC, were used to analyze ARPC5 expression in pan-cancer. The Human Protein Atlas website was applied to obtain the expression of ARPC5 in different tissues, cell lines, and single-cell types. Univariate Cox regression analysis and Kaplan–Meier analysis were used to explore the prognosis value of ARPC5 in various cancers. Spearman’s correlation analysis was performed to investigate the association between ARPC5 expression and tumor microenvironment scores, immune cell infiltration, immune-related genes, TMB, MSI, RNA modification genes, DNA methyltransferases, and tumor stemness. Moreover, qPCR, Western blot, and immunohistochemistry were carried out to examine the differential expression of ARPC5 in HCC tissues and cell lines. CCK8, EdU, flow cytometry, wound-healing assays, and transwell assays were conducted to explore its role in tumor proliferation, apoptosis, migration, and invasion among HCC cells.

**Results:**

ARPC5 expression was upregulated in most cancer types and significantly associated with worse prognosis in KIRC, KIRP, LGG, and LIHC. mRNA expression of ARPC5 showed low tissue and cell specificity in normal tissues, cell lines, and single-cell types. ARPC5 expression was positively correlated with the tumor microenvironment scores, immune infiltrating cells, immune checkpoint–related genes in most cancers. ARPC5 in STAD and BRCA was positively associated with TMB, MSI, and neoantigens. We also discovered that ARPC5 was correlated with the expression of m1A-related genes, m5C-related genes, m6A-related genes, and DNA methyltransferases. In experiment analyses, we found that ARPC5 was significantly highly expressed in HCC tissues and HCC cells. Functionally, silencing ARPC5 dramatically decreased proliferation, migration, and invasion ability of HCC cells.

**Conclusions:**

ARPC5 expression affects the prognosis of multiple tumors and is closely correlated to tumor immune infiltration and immunotherapy. Furthermore, ARPC5 may function as an oncogene and promote tumor progression in HCC.

## Introduction

Actin-related protein 2/3 complex (Arp2/3) is one of the major molecules that promotes the nucleation of new microfilaments and generates branched actin networks in the process of actin protein assembling into microfilaments (1). Arp2/3 complex is composed of seven conserved subunits: two actin-like subunits (Arp2 and Arp3) and four structural subunits (ARPC1/p40, ARPC2/p34, ARPC3/p21, ARPC4/p20, and ARPC5/p16) (2). The conformation of these subunits is changed by regulatory activators and inhibitory proteins; the activated Arp2/3 complex contributes to the actin-branched junction and, thus, cross-links the polymerizing actin filaments (1). As an inseparable element in the context of the actin cytoskeleton, Arp2/3 complex has been proved involving in many essential functions, including cell division, adhesion, migration, and endocytosis (3). The invasion and metastasis of cancer cells are mainly relied on actin-related pseudopodia, microfilaments, and associated proteins. The overactivation of the Arp2/3 complex generally increases the formation of invasive pseudopodia and, thus, promotes cancer migration and metastasis (4). Previous studies found that Arp2/3 subunits are highly expressed in a variety of cancers and promote the tumorigenesis and development, including pancreatic cancer (5, 6), breast cancer (7–9), lung squamous cell carcinoma (10), prostate cancer (11), gastric cancer (12), colorectal cancer (2), and bladder cancer (4). Despite the vital role of Arp2/3 complex in an extensive range of cellular processes, studies on the specific functions and mechanisms of some subunits in the complex are relatively scarce, including ARPC5.

ARPC5 is a core component of actin-related protein 2/3 (Arp2/3) complex, which is essential for activating Arp2/3 complex-mediated actin nucleation. The abnormal expression of ARPC5 likely causes functional aberrations of the whole complex. Several studies demonstrated that ARPC5 contributes to tumor growth or metastasis, including head and neck squamous cell carcinoma (13), lung squamous cell carcinoma (10), and melanoma (14). Moreover, bioinformatics analyses have suggested that ARPC5 expression is significantly increased in multiple myeloma (MM) cells compared with normal plasma cells, and high expression of ARPC5 is associated with poor overall survival (OS) in patients with MM. Our previous study also suggested that the higher ARPC5 expression has significantly poor OS and acts as an independent factor in predicting poor prognosis of hepatocellular carcinoma (HCC) patients (15). Nevertheless, the expression pattern, prognosis values, and biological roles of ARPC5 in most types of cancer have seldomly been analyzed systematically. Thus, it is essential to explore the roles of ARPC5 in pan-cancer from a novel and comprehensive perspective.

In this study, we conducted pan-cancer analyses of ARPC5 among 33 human cancer types using the Cancer Genome Atlas (TCGA) datasets, Genotype-Tissue Expression (GTEx) datasets, and some online bioinformatic analysis websites. We first investigated the expression pattern of ARPC5 in pan-cancer and discussed the associations of ARPC5 expression with pan-cancer prognosis and clinicopathological parameters. We also explored the correlation between ARPC5 and tumor microenvironment (TME) scores, immune cell infiltration, and immune subtypes. Moreover, the association between ARPC5 and tumor immunotherapy response was unveiled. In addition, a series of experiments were conducted to confirm the differential expression of APRC5 in HCC cell lines and HCC tissues and explore its potential biological functions in HCC cells. Our study preliminarily revealed the latent application of the ARPC5 as a predictive biomarker of prognosis and immunotherapy response in pan-cancer, which deserves further research.

## Materials and methods

### Clinical samples and ethnics approval

A total of 40 paired pathologically diagnosed HCC specimens and adjacent normal liver tissues were obtained after liver resection at the Second Affiliated Hospital of Nanchang University (Nanchang, China) from November 2020 to November 2021. One part of the specimens was fixed with 10% formalin, others were frozen with liquid nitrogen and stored in −80°C freezer until further processing. This study was prior approved by The Second Affiliated Hospital of Nanchang University Medical Research Ethics Committee, and written informed consent was provided by each patient enrolled in this study in accordance with the Declaration of Helsinki. Clinical and pathological characteristics of each patient were collected and shown in **Table 1**. We also obtained the postsurgical Disease-Free Survival (DFS) data of all participants until July 2022 (last follow-up visit).

**Table 1:** Clinical and pathological features of HCC patients.

Characteristics	Number of cases (%)
**Age**	
≤60	27 (67.5)
>60	13 (32.5)
**Gender**	
Male	36 (90)
Female	4 (10)
**HBsAg**	
Negative	9 (22.5)
Positive	31 (77.5)
**Child-Pugh classification**	
A	21 (52.5)
B	19 (27.5)
**AFP**	
≤400 ng/ml	25 (62.5)
>400 ng/ml	15 (37.5)
**Liver cirrhosis**	
Absent	9 (22.5)
Present	31 (77.5)
**Tumor number**	
Single	30 (75)
Multiple	10 (25)
**Lymph nodes metastasis**	
N0	37 (92.5)
N1	3 (7.5)
**Distant metastasis**	
M0	40 (100)
M1	0 (0)
**Edmondson–Steiner grades**	
I	3 (7.5)
II	20 (50)
III	19 (47.5)
IV	0 (0)

### Acquisition and processing public sequencing data of pan-cancer

The RNA-sequencing data of pan-cancer (33 cancer types) were downloaded from the UCEC database (http://xena.ucsc.edu/), which integrated TCGA database and GETx Project. The datasets were normalized and batched to the log_2_ (Fregments Per Kilobase per Million [FPKM]+1). Five cancers with less than three samples were eliminated, and the remaining 28 cancer types were involved in the gene differential analysis. Wilcoxon Rank Sum Test was used to evaluate the ARPC5 expression level between tumor tissues and the unpaired or paired normal tissues using the “ggplot2”and “reshape2” package of R 4.0.5 software (http:///www.r-project.org/), a value of *p* < 0.05 was considered to be statistically significant. Afterward, mRNA expression of ARPC5 in different tissues, cell lines, and single-cell types were directly obtained from the Human Protein Atlas website (https://www.proteinatlas.org/).

### Genetic mutation analysis of ARPC5 in pan-cancer

The web-accessible database cBioPortal (https://www.cbioportal.org/) was utilized to analyze the gene mutation characteristics of ARPC5, including the alteration frequency, mutation type, and copy number alteration in pan-cancer. The results were presented by pressing “quick search,” entering ARPC5, and selecting”Cancer Types Summary” model. Then, to further explore the correlation between ARPC5 expression and genomic variation, first, we downloaded copy number variation (CNV) datasets of the levels 4 processed by GISTIC software from TCGA database and integrated the CNV data with gene expression data. Next, Wilcoxon Rank Sum Test or Kruskal–Wallis Rank Sum Test was conducted to investigate the ARPC5 differential expression in different CNV subgroups of the pan-cancer. *p*-values of less than 0.05 (*p* < 0.05) were considered significant.

### The correlation analysis of ARPC5 with prognosis and clinical characteristics in pan-cancer

First, the clinical information and prognosis data were acquired from the UCEC database (http://xena.ucsc.edu/), which derived from a TCGA prognosis study (16), including OS, progression-free interval (PFI), and disease-specific survival (DSS). Then, univariate Cox regression models and Kaplan–Meier analysis were conducted to explore the relationship between ARPC5 expression and prognosis in pan-cancer *via* using “survival” and “survminer” R package. The significance was obtained *via* Log-rank statistical test between the high- and low-expression subgroups. The statistical significance was defined as *p* < 0.05. Thereafter, TISIDB (http://cis.hku.hk/TISIDB/index.php) (17), which is a web portal for tumor and immune system interaction and contains numerous heterogeneous data types from TCGA database, was used to explore the correlation between ARPC5 expression and pan-cancer clinical stages, histologic grades, and tumor molecular subtypes. Correlations were assessed using Spearman’s correlation analysis and presented as rank coefficient (rho) and *p*-value. The results were exhibited when *p*-values were < 0.05.

### The correlation of ARPC5 expression and TME and tumor immunity

The TME significantly influences the progression and metastasis of tumors, in which immune and stromal cells are two major non-tumor components (18). The Immune and Stromal scores were calculated by ESTIMATE algorithm using the “estimate” R package, which respectively represent the proportion of immune cells and stromal cells in the TME of tumor samples. Then, we performed the Spearman’s correlation analysis to evaluate the association between ARPC5 expression and the Immune/Stromal scores.

Thereafter, Tumor Immune Evaluation Resource (TIMER) (http://timer.comp-genomics.org/), a web server for comprehensive analysis of tumor-infiltrating immune cells, was used to calculate the infiltration scores of B cell, CD4 T cell, CD8 T cell, neutrophil, macrophage, and dendritic cells (DCs) in each sample. We selected the “Gene” module in TIMER and applied Spearman’s correlation analysis to assess the correlation between the expression of ARPC5 and immune cell infiltration. A *p* value < 0.05 was considered statistically significant. The results were presented with heatmap, and the top five cancer types with the strongest correlations were displayed with scatterplots.

Immune subtypes can effectively characterize intra-tumoral immune states, including six immune subtypes: C1 (wound healing), C2 (IFN-gamma dominant), C3 (inflammatory), C4 (lymphocyte depleted), C5 (immunological quiet), and C6 (TGF-beta dominant) (19). Different tumor types varied substantially in their proportion of immune subtypes. To identify the relationship between the expression of ARPC5 and immune subtypes in different cancer types, we applied online TISIDB web portal and the Kruskal–Wallis Test to conduct the differential expression analysis of ARPC5 in different immune subtypes of pan-cancer. Significance for the results was established and displayed when *p*-values were < 0.05.

### The relationship between the ARPC5 expression and immunotherapy

Immunotherapy is a validated and critically important approach for treating patients with cancer (20). In recent years, immune checkpoint inhibitors (ICIs) have shown remarkable potential in several types of cancer (21). The expression profiling of immune checkpoint–related genes on tumor cells or immune cells might effectively predict clinical benefit to checkpoint inhibitor strategies (22). Moreover, numerous studies have proved that tumor mutation burden (TMB), microsatellite instability (MSI), and neoantigens produced by somatic mutations were primary drivers of tumor immune responses, and mutational or neoantigen burden has also been studied as a predictive biomarker in patients given checkpoint inhibitors (22, 23). In this study, we first acquired the gene mutation data of 33 cancer types possessed with “varscan 2” method from TCGA database and calculated the TMB of each cancer sample with Perl 5.30.0 software (https://www.perl.org/). Meanwhile, we obtained summarized MSI data of pan-cancer from previous studies (24, 25). Then, we probed the association between ARPC5 expression with 47 immune checkpoint–related genes, TMB, and MSI with Spearman’s correlation method. Moreover, Sangerbox website (http://sangerbox.com) was utilized to investigate the correlation between ARPC5 expression and neoantigens *via* “Tool” module and Spearman’s correlation test. All the results were visualized as heatmaps or radar plots.

### Correlation analysis of ARPC5 expression with RNA modification, DNA methylation, and tumor stemness

Increasing evidences indicated that RNA modification pathways are misregulated in human cancers and closely connected to cancer pathogenesis. Of those, the common and characterized RNA modification are the methylation of adenosine at position 6 to give N6­methyladenosine (m6A), RNA 5­methylcytosine (m5C), and methylation of adenosine at position 1 to give N1­methyladenosine (m1A) (26). Thus, we performed Spearman’s correlation analysis to explore the relationship of ARPC5 expression with the three types of RNA modification related genes, including 10 m1A-related genes (TRMT61A, TRMT61B, TRMT10C, TRMT6, YTHDF2, YTHDF3, YTHDF1, YTHDC1, ALKBH1, and ALKBH3), 13 m5C-related genes (TRDMT1, NSUN3, NSUN4, NSUN5, NSUN7, DNMT3A, NSUN2, DNMT1, NSUN6, NOP2, DNMT3B, TET2, and ALYREF), and 21 m6A-related genes (VIRMA,WTAP, METTL14, CBLL1, RBM15, METTL3, RBM15B, ZC3H13, ALKBH5, FTO, IGF2BP1, LRPPRC, FMR1, YTHDC1, YTHDC2, HNRNPC, YTHDF2, YTHDF3, YTHDF1, HNRNPA2B1, and ELAVL1).

DNA methylation is an important epigenetic modification regulating gene expression, and deregulation of DNA methylation is strongly associated with the tumor occurrence and development (27). The process of DNA methylation is regulated by different DNA methyltransferase enzymes. In our study, we analyzed the relationship between ARPC5 and DNA methylation process by evaluating the co-expression association of five methyltransferases (DNMT1, TRDMT1, DNMT3A, DNMT3B, and DNMT3L) and ARPC5.

Cancer progression involves in gradual loss of differentiated phenotype and acquisition of stem cell–like features. A great number of genomic, epigenomic, transcriptomic, and proteomic signatures have been associated with cancer stemness (23). In this study, we obtained tumor stemness scores (DNAss and RNAss) calculated by DNA methylation signature and mRNA expression from previous study (28) and integrated transcription expression data with two stemness scores to perform the Spearman’s correlation test. The online website Sangerbox was used to explore the correlation between ARPC5 expression and stemness indexes of pan-cancer.

### Cell lines and RNA interference

The HCC cell lines MHCC 97-H, Huh7, HCC-LM3, and HepG2 were purchased from Procell (Wuhan, China). The normal liver cell L-02 was obtained from the Chinese Academy of Science. These cells were maintained in Dulbecco’s modified Eagle’s medium (DMEM; Solaibio, Beijing, China) supplementing with 10% fetal bovine serum (FBS) (Gibco, Grand Island, NY, USA), 100 µg/ml streptomycin and 100 U/ml penicillin sodium (Biotechnology, Beijing, China) at 37°C.

Three different small-interfering RNA (siRNA) sequences, targeted to ARPC5 and negative control siRNA, were designed and synthesized by GenePhram Gene (A09001, Shanghai, China). The siRNA fragments were transfected with TransIntroTM EL Transfection Reagent (TransGen Biotech, Beijing, China) in accordance with the manufacturer’s protocol. Transfected cells were cultured in DMEM medium without FBS and replaced with complete medium after 4–6 h. Subsequent experiments were conducted after transfection for 48 h. The sequences of siRNA-targeted ARPC5 were listed as follows: si-ARPC5#1 sense 5′- GUGGAUGAAUAUGACGAGATT-3′ and antisense 5′-UCUCGUCAUAUUCAUCCACTT-3′; si-ARPC5#2 sense 5′-GGCAUU CCAUCACAGGAAATT -3′ and antisense 5′- UUUCCUGUGA UGGAAUGCCTT -3′; si-ARPC5#3 sense 5′-GCAGUGCUAUGUUACU GCATT-3′ and antisense 5′- UGCAGUAACAUAGCACUGCT-3′; negative control: sense 5′- UUCUCCGAACGUGUCACGUTT-3′ and antisense 5′- ACGUGACACGUUCGGAGAATT-3′.

### Quantitative real-time PCR

Total RNA was extracted using Trizol Reagent (Invitrogen, Carlsbad, CA, USA) according to the manufacturer’s instructions. Next, the RNA was reversely transcribed to first-strand cDNA *via* the EasyScript^®^ One-Step gDNA Removal and cDNA Synthesis SuperMix (AE311-03, TransGen Biotech, Beijing, China). Then, quantitative real-time polymerase chain reaction (qPCR) was conducted with TB Green^®^ Premix Ex Taq™ II (RR820A, TaKaRa, China), taking Glyceraldehyde 3-phosphate dehydrogenase (GAPDH) as the endogenous control. The relative mRNA expression of HCC cells was calculated using the 2^-ΔΔCT^ method, and the relative mRNA expression of HCC tissues was reckoned by 2^-ΔCT^. The gene primers were presented as follows: ARPC5 Forward: 5′-TGGTGTGGATCTCCTAATGAAGT-3′; Reverse: 5′-CACGAACAATGG ACC CTACTC-3′; GAPDH Forward: 5′- GGAGCGAGATCCCTCCAAAAT-3′; Reverse: 5′- GGCTGTTGTCATA CTTCTCATGG-3′.

### Western blotting analysis

The total protein was extracted from the HCC cells and HCC tissues with radioimmunoprecipitation assay (RIPA) lysis buffer and protease inhibitor (Beyotime, Shanghai, China). Protein concentration was assessed by BCA assay kit (Beyotime, Shanghai, China). Proteins were separated in a 10% SDS-polyacrylamide gel electrophoresis (SDS-PAGE) and transferred to a PVDF membrane. The membrane was blocked with 5% nonfat milk for 2 h at room temperature and incubated with diluted primary antibody overnight at 4°C. The primary antibodies anti-ARPC5 was purchased from Abmart (1:2000, T553316S, Shanghai, China), and primary antibodies against GAPDH (1:5000, 60004-1-Ig), E-cadherin (1:5000, 20874-1-AP), N-cadherin (1:2000, 22018-1-AP), snail (1:1000,13099-1-AP), and vimentin (1:5000, 10366-1-AP) were purchased from Proteintech (Wuhan, China). Then, the membrane was treated with horseradish peroxidase (HRP)–labeled goat anti-rabbit or anti-mouse IgG antibodies (SA00001-1/SA00001-2, Proteintech, diluted at 1:10000) at room temperature for 1 h. The protein bands were detected using the chemiluminescence ELC (Beyotime, Shanghai, China) and Bio-Rad (Hercules, CA, USA) gel scanning. ImageJ software (ImageJ 1.8.0, NIH, Bethesda, MD) was used to quantitatively analyze the relative protein content.

### Immunohistochemistry staining

The 40-paired fresh HCC tissues and adjacent normal liver tissues were collected immediately after resection from the Second Affiliated Hospital of Nanchang University, formalin-fixed, paraffin-embedded, planked on a glass slide, and baked at 60°C for 2 h. This was followed with standard xylene dewaxed procedure, hydrated with the gradient ethanol, and blocked the endogenous peroxidases with 0.3% H_2_O_2_. After the antigen retrieval, the rabbit anti-human ARPC5(1:500, T553316S, Abmart) primary antibody was applied to the slides and incubated at 4°C overnight and followed by the secondary anti–horseradish peroxide for 30 min. Next, the slides were stained with DAB chromogenic reagent and hematoxylin. Finally, slides were dehydrated, transparent and sealed. Microscopic images were observed by light microscopy. Representative results were presented. The Image-pro plus 6.0 software (Media Cybernetics, Inc., Rockville, MD, USA) was used to calculate cumulative optical density (IOD) and pixel area of tissue, and the immunohistochemical results were expressed as mean optical density.

### Cell proliferation assays

CCK8 assays and 5-ethynyl-2′-deoxyuridine (EdU) staining assays were used to detect the proliferation ability of HCC cells. For the CCK8 assay, a total of 2 × 10^3^ transfected cells were uniformly seeded in 96-well plates and cultured for 12, 24, 48, and 72 h. CCK8 reagent (10 μl) was added into each well for 2-h incubation. The absorbance at a wavelength of 450 nm was detected on an enzyme immune-assay analyzer (Bio-Rad, Hercules, CA, USA). EdU assay was performed using YF 555 Click-iT EdU kit (C6016L, US Everbright^®^ Inc., China) according to the manufacturer’s instructions. The transfected HCC cells were planted in the 96-well plate, incubated for 24 h, and labeled with EdU reagent. After fixation and permeabilization, the cells were stained with EdU fluorescence staining kit. The images were observed and photographed by fluorescence microscopy, and ImageJ software was used to calculate the percentage of proliferation cells.

### Flow cytometry

After 48 h of transfection, cell apoptosis was detected with FITC-Annexin V/PI apoptosis detection kit (F6012, US Everbright^®^ Inc., China). According to the product instruction, first, the cells were digested with EDTA-free trypsin, centrifuged at 1,000 rpm/min for 5 min, washed three times with phosphate-buffered saline (PBS), and resuspended the cells with 100 µl of mixed buffer. Then, the cell samples were stained with 5 μl of PI and 5 μl of FITC-Annexin V. After incubating at 4°C for 15 min protected from light, another 400 μl of binding buffer was added to the flow samples and mixed well. At last, flow cytometer (FACSCalibur flow cytometer; BD Biosciences, San Jose, CA, USA) was used to detect the apoptotic cells, and the apoptosis percentage was calculated, including early apoptosis (Annexin V^+^/PI^-^) and late apoptosis (Annexin V^+^/PI^+^).

### Cell migration and invasion assays

The migration ability of HCC cells was detected by Scratch assays. First, the transfected HCC cells were evenly plated and incubated in 6-well until 100% confluence. Then, a sterile 100-μl pipette tip was used to scratch the cell monolayer and produce a clear wound. The cells were washed with PBS to remove floating cells and cultured with fresh serum containing medium for 48 h. The cells images were acquired with an optical microscope system at 0, 24, and 48 h. The scratch area was measured with ImageJ software, and cell mobility was determined with the following formula: Cell migration rate (%) = (1 − scratch area/original scratch area) × 100%.

The invasion ability of HCC cells was analyzed by the Transwell chamber assay. The upper chamber was pre-covered with a layer of Matrigel gel (YB356234, BD Biosciences, USA), placed into a 24-well plate and dried overnight. Then, the transfected cells (2 × 10^4^) were suspended with 200 μl of serum-free medium and seeded in the upper chamber, cell medium with 10% FBS was added to the lower chamber. After incubation for 48 h, the cells were fixed with 4% formaldehyde and stained with 0.1% crystal violet staining solution. The number of invading cells was counted using a light microscope at ×200 magnification.

### Statistical analysis

R software (https://www.r-project.org/version 4.0.4) was used to perform bioinformatic analyses. Wilcoxon Rank Sum Test or Kruskal–Wallis Rank Sum Test was applied to evaluate the differences between groups. Kaplan–Meier method and Cox regression analysis were used for survival analysis. Spearman’s correlation analyses were performed to clarify the correlations between groups. The experimental data were analyzed with GraphPad Prism 9.0 software. All experiments were repeated in triplicates. The results were reported as the *M* ± *SD*. The differences between groups were analyzed by using Student’s *t*-test or one-way analysis of variance (ANOVA). All statistical tests were two-sided, and statistical significance was set at *p* < 0.05.

## Results

### The expression analysis of ARPC5 in pan-cancer

**Figure 1:**
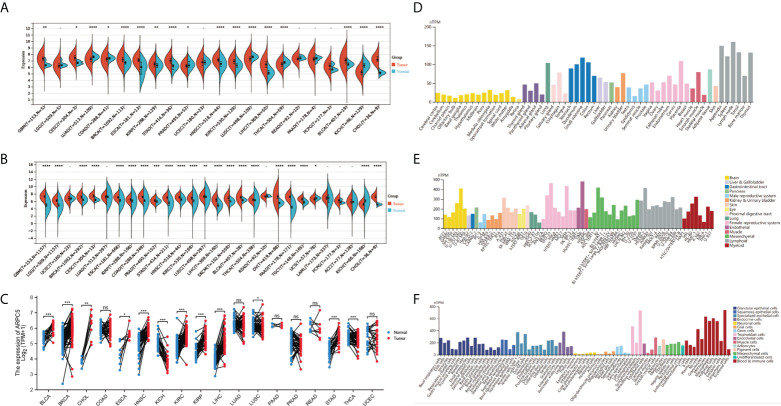
The expression levels of APRC5 in different cancers, normal tissues, and cells. **(A)** The differential expression of ARPC5 in pan-cancer tissues from TCGA datasets. **(B)** The differential expression of ARPC5 in pan-cancer tissues based on TCGA and GTEx datasets. **(C)** ARPC5 expression in paired cancer tissues and adjacent normal tissues from TCGA datasets. **(D)** The mRNA expression levels of ARPC5 in different normal tissues from HPA database. **(E)** The mRNA expression of ARPC5 in cancer cell lines from HPA database. **(F)** ARPC5 mRNA expression in different single cell types from HPA database. ns: no significance; *p < 0.05; **p < 0.01; ***p < 0.001; ****p < 0.0001.

In our study, we first downloaded the RNA-sequencing data of 33 cancer types from UCSC database basing on TCGA datasets and GETx datasets. After removing the cancer types with less than three samples, 22 cancer types of TCGA data (*N* = 8886) and 28 cancer types in TCGA target GTEx data (*N* = 16962) were enrolled in gene differential analysis. Then, the differential expression of ARPC5 between tumor and normal tissues of pan-cancer was assessed using Wilcoxon Rank Sum Test. **Figure 1A** showed the analysis of TCGA dataset, the mRNA expression of ARPC5 in tumor tissues of GBM, CESC, BRCA, ESCA, KIRP, STAD, HNSC, KIRC, LIHC, BLCA, and CHOL was higher than the corresponding normal tissues. While significant downregulation of ARPC5 was observed in LUAD, COAD, PRAD, LUSC, THCA, and KICH. After integrating the TCGA data with GTEx datasets, we discovered that the ARPC5 was upregulated in other 10 cancer types, including LGG, COAD, PRAD, LUSC, WT, SKCM, THCA, OV, PAAD, and TGCT. ARPC5 was downregulated in UCS, ALL, and KICH (**Figure 1B**). In addition, paired sample analysis was performed in the 18 cancer types based on TCGA datasets, ARPC5 expression was found to be upregulated in tumor tissues of BRCA, BLCA, CHOL, ESCA, HNSC, KIRC, KIRP, LIHC, LUSC, and STAD, while downregulated in KICH and THCA. (**Figure 1C**). The results indicated that the expression of ARPC5 was upregulated in most cancer types.

Furthermore, Human Protein Atlas website was applied to assess the APRC5 expression in different tissues and cell lines. As shown in **Figures 1D–F**, the ARPC5 expressed in all normal tissues and cell lines, showing low RNA tissue and cell specificity in human normal tissues, tumor cell lines, and single cell types. The mRNA expression levels of ARPC5 were relatively higher in lymph nodes, appendix, and blood and immune cells whereas lower in brain tissues and neuronal cells.

### Genetic alteration status of ARPC5 in pan-cancer

**Figure 2:**
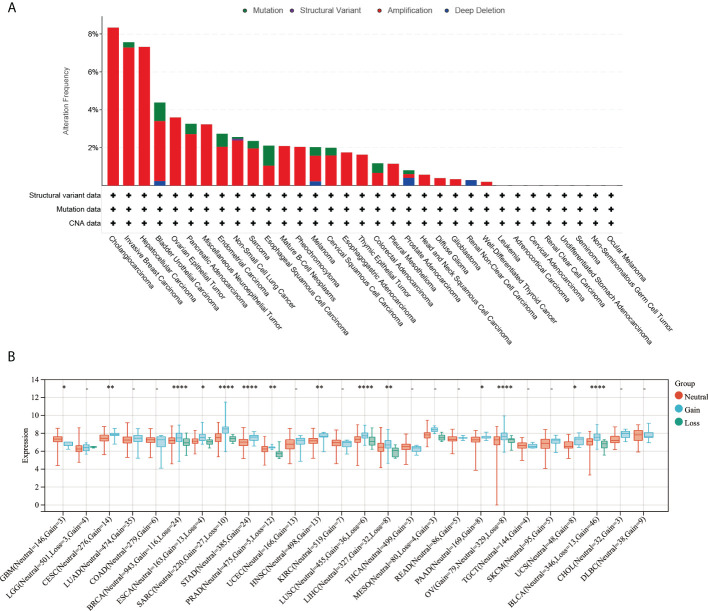
Genetic alteration of ARPC5 in pan-cancer. **(A)** Mutation type and mutation frequency of ARPC5 obtained from the cBioPortal website. **(B)** The expression levels of ARPC5 in various CNV status of pan-cancer, CNV, copy number variations; *p < 0 . 0 5 ; * *p < 0.01; ****p < 0.0001.

The online platform cBioPortal was used to analyze the gene alteration frequency and mutation type of ARPC5 in pan-cancer. The results indicated the most common alteration types was gene “Amplification,” followed by “Mutation,” “Deep Deletion,” and “Structural Variant.” The highest alteration frequency of ARPC5 was observed in cholangiocarcinoma, in which three of 32 cases (8.33%) happened gene “Amplification.” The gene alteration frequency was 7.56% in invasive breast carcinoma and 7.26% in HCC; the alteration frequency of other cancer types was less than 5% (**Figure 2A**). Moreover, we further explored the relationship between genomic variation and ARPC5 expression in pan-cancer *via* integrating CNV and gene expression data. Wilcoxon Rank Sum Tests or Kruskal–Wallis Rank Sum Test was used to compare the expression levels of ARPC5 in different variation status of each cancer. The results were shown in **Figure 2B**, remarkable difference of ARPC5 expression was found among gain variation, loss variation, and no variation groups in 14 cancer types, such as GBM, CESC, BRCA, ESCA, SARC, STAD, PRAD, HNSC, LUSC, LIHC, PAAD, OV, UCS, and BLCA. Taking LIHC as an example, neutral group showed higher ARPC5 expression than gain and loss variation groups. That indicating ARPC5 expression was closely associated with the mutation type in multiple cancer types.

### The correlation of ARPC5 expression with prognosis and clinicopathology features in pan-cancer

**Figure 3:**
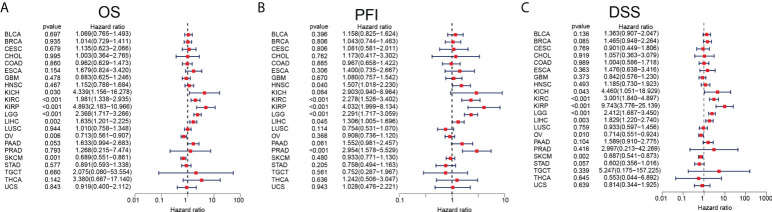
Prognosis analyses of ARPC5 in pan-cancer based on univariate Cox regression method. **(A)** The correlation between ARPC5 expression and OS. **(B)** The correlation between ARPC5 expression and PFI. **(C)** The correlation between ARPC5 expression and DSS. OS: overall survival; PFI: progression-free interval; DSS: disease-specific survival.

**Figure 4:**
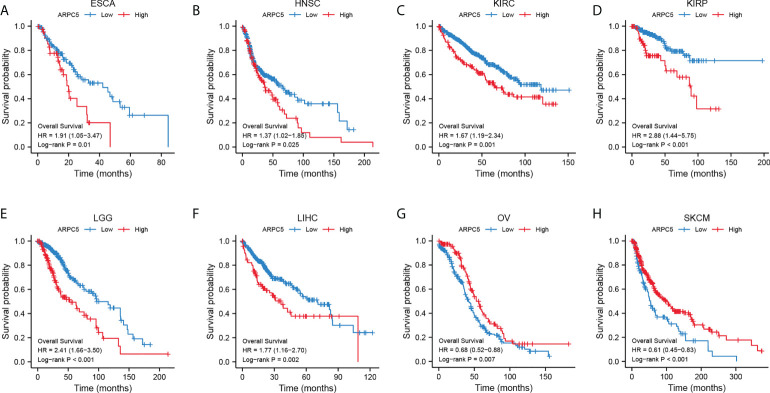
ARPC5 expression significantly correlated with OS based on Kaplan–Meier analysis. **(A)** The correlation in ESCA. **(B)** The correlation in HNSC. **(C)** The correlation in KIRC. **(D)** The correlation in KIRP. **(E)** The correlation in LIHC. **(F)** The correlation in LGG. **(G)** The correlation in OV. **(H)** The correlation in SKCM. The optimal cutoff of ARPC5 expression were used to divide patients into high- and low-expression groups.

We had clarified that ARPC5 was significantly differentially expressed among 22 cancer types in above analysis. To further investigate the correlation between the expression of ARPC5 and cancer prognosis (including OS, PFI, and DSS), single-variate Cox regression method and Kaplan–Meier analysis were conducted in 22 cancers. For univariate Cox regression analysis (**Figures 3A–C**), the results demonstrated that the higher ARPC5 expression was associated with worse OS in KICH, KIRC, KIRP, LGG, and LIHC, whereas the opposite results were observed in patients with OV and SKCM. The results of PFI showed higher ARPC5 expression related to shorter PFI in HNSC, KIRC, KIRP, LGG, LIHC, and PRAD. Moreover, the expression level of ARPC5 was negatively linked with DSS in KICH, KIRP, KIRC, LGG, and LIHC, whereas positive association was found in OV and SKCM. Kaplan–Meier analysis and Log-rank test further proved that high ARPC5 expression was correlated to worse OS in ESCA, HNSC, KIRC, KIRP, LGG, and LIHC, whereas the opposite results were observed in OV and SKCM (**Figures 4A–H**). The PFI results of Log-rank test indicated that expression of ARPC5 was negatively correlated with PFS in patients with BLCA, HNSC, KIRC, KIRP, LGG, LIHC, and PRAD (**Supplementary Figure S1**). The DSS results of Kaplan–Meier analysis manifested that ARPC5 expression adverse to DSS in patients with BLCA, KIRC, KIRP, LCC, and LIHC, whereas positively correlated to DSS of LUSC, OV, SKCM, and STAD (**Supplementary Figure S2**). In brief, these results suggested the ARPC5 can serve as an effective prognosis predictor in multiple cancers.

**Figure 5:**
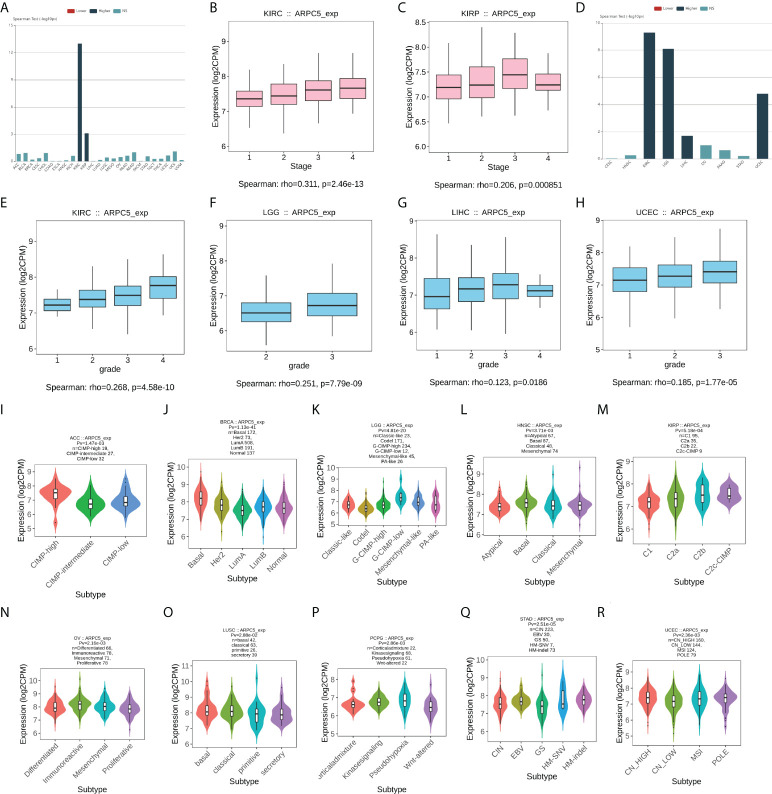
The correlation between ARPC5 expression and clinical stage, histologic grade, and tumor molecular subtypes in various cancers based on Spearman’s correlation analysis (the correlation with *p* < 0.05 were displayed). **(A)** The correlation between ARPC5 expression and clinical stage in pan-cancer. **(B–C)** The expression levels of ARPC5 in different clinical stages of KIRC **(B)** and KIRP **(C)**. **(D)** The correlation between ARPC2 expression and histologic grade in pan-cancer. **(E–H)** The expression levels of ARPC5 in different histologic grades of KIRC **(E)**, LGG **(F)**, LIHC **(G)**, and UCEC **(H)**. **(I–R)** The correlation between ARPC5 expression and molecular subtypes in ACC **(I)**, BRCA **(J)**, LGG **(K)**, HNSC **(L)**, KIRP **(M)**, OV **(N)**, LUSC **(O)**, PCPG **(P)**, STAD **(Q)**, UCEC **(R)**. rho; rank coefficient of Spearman. Pv; *p*-value. NS, no significance.

We further explored the relationship between expression of ARPC5 and clinicopathology features in pan-cancer, including the clinical stage, histologic grade, and tumor molecular subtype. The results with significant association were selected for display and analysis (**Figure 5**). The results indicated the increased expression of ARPC5 was positively correlated to tumor stage of KIRC (ρ = 0.311, *p* = 2.46e-13) and KIRP (ρ = 0.206, *p* = 8.51e-04) (**Figures 5A–C**). Similarly, there was a positive correlation between ARPC5 expression and histologic grade of KIRC (ρ = 0.268, *p* = 4.58e-10), LGG (ρ = 0.251, *p* = 7.79e-09), LIHC (ρ = 0.123, *p* = 0.0186), and UCEC (ρ = 0.185, *p* = 1,77e-05) (**Figures 5D–H**). It, therefore, can be concluded that ARPC5 may associate with tumorigenesis and cancer progression. As for tumor molecular subtype, we found that the expression level of ARPC5 varied among different molecular subtypes of ACC (*p* = 1.47e-03), BRCA (*p* = 1.13e-41), LGG (*p* = 4.81e-20), HNSC (*p* = 3.71e-03), KIRP (*p* = 5.18e-04), OV (*p* = 2.16e-03), LUSC (*p* = 2.88e-02), PCPG (*p* = 2.86e-03), STAD (*p* = 2.51e-05), and UCEC (*p* = 2.36e-03) (**Figures 5I–R**).

### APRC5 expression is correlated with TME scores and immune infiltration levels in multiple cancers

In above analyses, we discovered the mRNA expression level of ARPC5 were relatively higher in immune cells. Thus, to further elucidate the potential impact of ARPC5 on the tumor immunity, we first applied the ESTIMATE algorithm to assess Immune and Stromal scores for 33 cancer types and analyzed the association between ARPC5 expression and Immune/Stromal scores using Spearman’s correlation method. As shown in **Table 2**, the expression of ARPC5 was evidently positively related to Immune scores in 22 cancer types, including BLCA, BRCA, COAD, GBM, HNSC, KICH, KIRC, KIRP, LAML, LGG, LUAD, LUSC, OV, PCPG, PRAD, SARC, SKCM, STAD, TGCT, THCA, THYM, and UCS. No significant differences were detected in other cancer types. The Stomal scores in 15 out of 33 cancers showed significantly positive correlation with ARPC5 expression, including BLCA, GBM, KICH, KIRC, KIRP, LAML, LGG, LUAD, LUSC, OV, PCPG, PRAD, SKCM, TGCT, and THCA. ARPC5 expression was negatively correlated with Stromal scores in THYM.

**Table 2:** The correlation between ARPC5 expression and immune scores and stromal scores of tumor microenvironments in pan-cancer.

Cancer types	Immune score		Stromal score	
	R	*p*-value	R	*p*-value
ACC	0.11	3.48E-01	0.15	1.73E-01
BLCA	0.23	**2.24E-06**	0.16	**1.34E-03**
BRCA	0.22	**2.50E-13**	0.026	3.95E-01
CESC	0.082	1.54E-01	0.037	5.15E-01
CHOL	0.083	6.29E-01	0.11	5.14E-01
COAD	0.13	**5.07E-03**	0.087	6.05E-02
DLBC	0.23	1.22E-01	0.25	8.98E-02
ESCA	0.013	8.70E-01	0.016	8.37E-01
GBM	0.48	**6.27E-11**	0.48	**9.12E-11**
HNSC	0.1	**1.97E-02**	-0.06	1.72E-01
KICH	0.56	**1.94E-06**	0.62	**7.97E-08**
KIRC	0.55	**<2.2E-16**	0.52	**<2.2E-16**
KIRP	0.42	**4.19E-14**	0.4	**1.38E-12**
LAML	0.57	**<2.2E-16**	0.65	**<2.2E-16**
LGG	0.6	**<2.2E-16**	0.56	**<2.2E-16**
LIHC	0.045	3.85E-01	0.0038	9.41E-01
LUAD	0.23	**1.31E-07**	0.24	**1.57E-08**
LUSC	0.24	**8.32E-08**	0.16	**3.72E-04**
MESO	-0.079	4.67E-01	-0.12	2.61E-01
OV	0.18	**3.20E-04**	0.19	**2.52E-04**
PAAD	0.092	2.24E-01	0.083	2.71E-01
PCPG	0.29	**9.05E-05**	0.4	**2.31E-08**
PRAD	0.43	**<2.2E-16**	0.39	**<2.2E-16**
READ	0.043	5.82E-01	0.081	3.01E-01
SARC	0.26	**2.08E-05**	0.12	5.64E-02
SKCM	0.19	**4.05E-05**	0.13	**5.93E-03**
STAD	0.11	**3.30E-02**	-0.031	5.48E-01
TGCT	0.54	**<2.2E-16**	0.41	**9.87E-08**
THCA	0.38	**<2.2E-16**	0.36	**<2.2E-16**
THYM	0.5	**1.15E-08**	-0.22	**1.62E-02**
UCEC	0.004	9.17E-01	-0.027	5.24E-01
UCS	0.4	**2.40E-03**	0.12	3.84E-01
UVM	0.059	6.04E-01	0.037	7.45E-01

R represents the coefficient correlation of Spearman analysis. Boldness indicates p-value less than 0.05.

**Figure 6:**
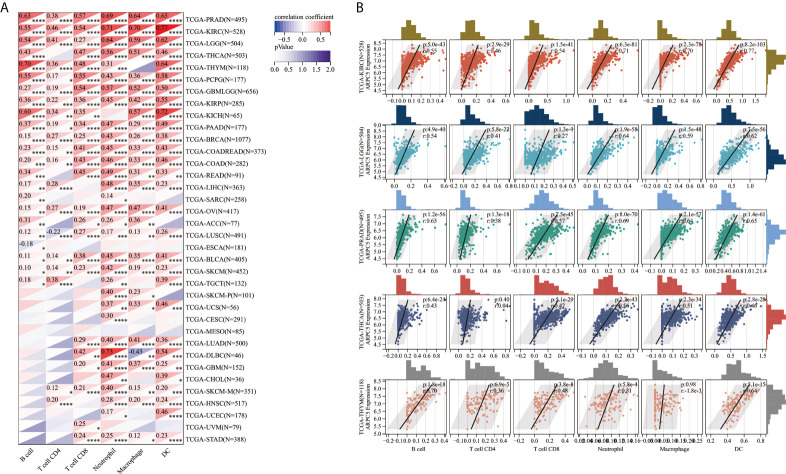
The correlation between ARPC5 and immune infiltration cells in pan-cancer based on TIMER algorithm. **(A)** Heatmap displayed the correlation between ARPC5 expression and the proportions of B cell, CD4^+^ T cell, CD8^+^ T cell, neutrophil, macrophage, and DC cell. **(B)** The top five cancer types (including KIRC, LGG, PRAD, THCA, and THYM) with most significant correlation between ARPC5 and immune infiltration cells were displayed with scatterplots. *p < 0.05; **p < 0.01; ***p < 0.001; ****p < 0.0001.

Moreover, TIMER database was used to explore the correlation of ARPC5 expression with infiltration levels of B cell, CD4^+^ T cell, CD8^+^ T cell, neutrophil cell, macrophage cell, and DC cell in pan-cancer. The results of the Spearman’s correlation analysis suggested that ARPC5 were positively associated with immune infiltration cells in most tumors. ARPC5 expression was significantly positively related to B cell in 22 cancer types, and negatively related to B cell in ESCA. The expression of ARPC5 was significantly associated with CD4^+^ T cell in 20 cancers, CD8^+^ T cell in 25 cancers, neutrophil cell in 32 cancers, macrophage cell in 27 cancers, and DC cell in 29 cancer types (**Figure 6A**). Among them, ARPC5 expression in KIRC, LGG, PRAD, THCA, and THYM was most closely related immune cells infiltration, the results were presented in **Figure 6B**. In KIRC, the expression of ARPC5 positively corresponded with the infiltration levels of B cell (*r* = 0.55, *p* = 5.0e-43), CD4^+^ T cell (*r* = 0.46, *p* = 2.9e-29), CD8^+^ T cell (*r* = 0.54, *p* = 1.5e-41), neutrophil cell (*r* = 0.71, *p* = 6.3e-81), macrophage cell (*r* = 0.7, *p* = 2.3e-78), and DC cell (*r* = 0.77, *p* = 8.2e-103). In LGG, ARPC5 expression had a positive correlation with the proportion of B cell (*r* = 0.54, *p* = 0.9e-40), CD4^+^ T cell (*r* = 0.41, *p* = 5.8e-22), CD8^+^ T cell (*r* = 0.27, *p* = 1.3e-09), Neutrophil cell (*r* = 0.64, *p* = 1.9e-58), macrophage cell (*r* = 0.59, *p* = 1.5e-48), and DC cell (*r* = 0.62, *p*=7.5e-56). In PRAD, the infiltrating levels of B cell (*r* = 0.63, *p* = 1.2e-56), CD4^+^ T cell (*r* = 0.38, *p* = 1.3e-18), CD8^+^ T cell (*r* = 0.57, *p* = 7.5e-45), neutrophil cell (*r* = 0.69, *p* = 8.0e-70), macrophage cell (*r* = 0.64, *p* = 2.1e-57), and DC cell (*r* = 0.65, *p* = 1.4e-61) were positively related to the expression of ARPC5. Similarly, ARPC5 expression had a positive association with the infiltration levels of B cell (*r* = 0.43, *p* = 6.4e-24), CD8^+^ T cell (*r* = 0.47, *p* = 5.1e-29), neutrophil cell (*r* = 0.56, *p* = 2.2e-43), macrophage cell (*r* = 0.51, *p* = 2.3e-34), and DC cell (*r* = 0.46, *p* = 2.8e-28) in THCA. In addition, ARPC5 positively related to the content of B cell (*r* = 0.7, *p* = 1.8e-18), CD4^+^ T cell (*r* = 0.36, *p* = 6.9e-5), CD8^+^ T cell (*r* = 0.48, *p* = 3.8e-8), neutrophil cell (*r* = 0.31, *p* = 5.8e-4), and DC cell (*r* = 0.64, *p* = 5.1e-15) in THYM.

**Figure 7:**
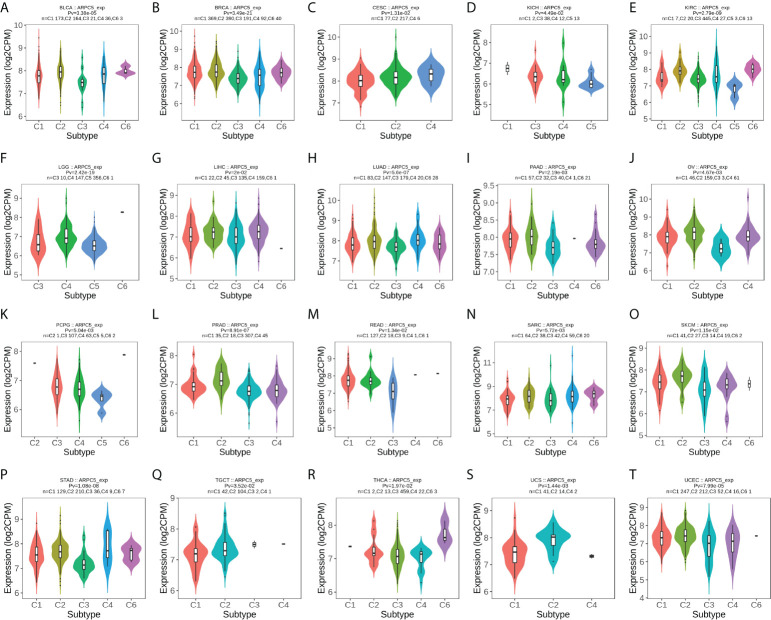
The correlation between ARPC5 expression and immune subtypes in pan-cancer using TISIDB. The cancers with significant correlation were displayed. **(A)** In BLCA. **(B)** In BRCA. **(C)** In CESC. **(D)** In KICH. **(E)** In KIRC. **(F)** In LGG. **(G)** In LIHC. **(H)** In LUAD. **(I)** In PAAD. **(J)** In OV. **(K)** PCPG. **(L)** PRAD. **(M)** In READ. (**N)** In SARC. **(O)** In SKCM. **(P)** In STAD. **(Q)** In TGCT. **(R)** In THCA. **(S)** In UCS. **(T)** In UCEC. Pv; *p*-value. C1, wound healing; C2, IFN-gamma dominant; C3, inflammatory; C4, lymphocyte depleted; C5, immunologically quiet; C6, TGF-b dominant.

In addition, we further probed into the relevance of ARPC5 expression with different immune subtypes of pan-cancer. The results revealed that ARPC5 expression was prominently related to immune subtypes in a number of cancers(**Figures 7A–T**), which included BLCA (*p* = 3.38e-05), BRCA (*p* = 3.49e-21), CESC (*p* = 1.31e-02), KICH (*p* = 4.49e-02), KIRC (*p* = 2.79e-09), LGG (*p* = 2.42e-19), LIHC (*p* = 2e-02), LUAD (*p* = 5.6e-07), PAAD (*p* = 2.19e-03), OV (*p* = 4.67e-03), PCPG (*p* = 5.04e-03), PRAD (*p* = 8.91e-07), READ (*p* = 1.34e-02), SARC (*p* = 5.72e-03), SKCM (*p* = 1.15e-02), STAD (*p* = 1.08e-08), TGCT (*p* = 3.52e-02), THCA (*p* = 1.97e-02), UCS (*p* = 1.44e-03), and UCEC (*p* = 7.99e-05). ARPC5 expression was generally low in C3 subtype, except for KICH, PCPG, TGCT, and ARPC5 was widely highly expressed in C2 subtype of 11 cancer types except CESC, KICH, LGG, and READ. Therefore, we speculated that ARPC5 might be more participant in IFN-gamma dominant immune processes but less involved inflammatory processes.

### The association between ARPC5 expression and immune-checkmate inhibitors biomarkers

**Figure 8:**
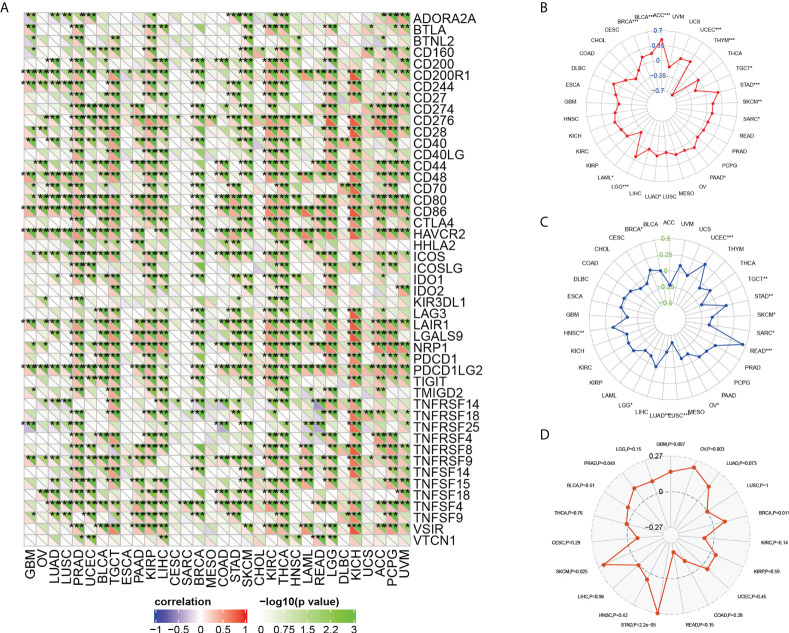
The relationship between ARPC5 and immune-checkmate inhibitors biomarkers in pan-cancer. **(A)** The heatmap showing the co-expression relationship between ARPC5 and 47 immune checkpoint–related genes. **(B)** Radar plot showing the relationship between ARPC5 and tumor mutation burden (TMB). **(C)** Radar plot showing the correlation of ARPC5 with microsatellite instability (MSI). **(D)** Radar plot showing the correlation of ARPC5 with neoantigens. The number in radar plot represents Spearman’s correlation coefficient. *p < 0.05; **p < 0.01; ***p < 0.001.

Previous studies have proved that ICIs-related genes, TMB, MSI, and tumor neoantigens can be used as effective predictors of ICIs. Thus, we discussed the correlations of ARPC5 expression with these ICIs biomarkers. First, Gene co-expression and Spearman’s coefficient analyses were conducted to investigate the association between ARPC5 expression and 47 ICIs-related genes in 33 cancer types. We discovered ARPC5 was closely related to the expression of ICIs-related genes in most types of cancer, such as PRAD, TGCT, KIRC, LIHC, KIRC, THCA, LGG, KICH, PCPG, and so on. However, there was less association between ARPC5 and ICIs-related genes in CESC, SARC, MESO, and UCS (**Figure 8A**). In LIHC, ARPC5 exhibited significant positive correlation with most ICIs-related genes. That indicated that the ARPC5 may act as a new biomarker for ICIs in LIHC or other certain cancers.

Then, we performed an exploration to analyze the relationship of ARPC5 with TMB and MSI by integrating gene expression data and TMB and MSI data. The results of Spearman analysis showed that the expression level of ARPC5 was positively related to TMB in ACC, UCEC, STAD, SKCM, SARC, PAAD, LUAD, LGG, BRCA, and BLCA, whereas reverse correlation was presented in THYM, TGCT, and LAML (**Figure 8B**). We also found that the expression of ARPC5 was significantly related to MSI in 12 types of cancer. Of those, positive correlations were detected in BRCA, UCEC, STAD, READ, and HNSC; negative relations were observed in TGCT, SKCM, SARC, OV, LUSC, LUAD, and LGG (**Figure 8C**).

In addition, tumor neoantigens are abundantly expressed in tumor cells with strong immunogenicity and tumor heterogeneity (22). Therefore, we further measured the correlation between ARPC5 expression and tumor neoantigens. As shown in **Figure 8D**, the tumor neoantigens in five of 19 cancers showed significant positive correlation with APRC5 expression, including OV (*p* = 0.003), BRCA (*p* = 0.011), STAD (*p* = 2.2e-05), SKCM (*p* = 0.025), and PRAD (*p* = 0.049).

### ARPC5 correlated with RNA modification-related genes, DNA methyltransferases, and tumor stemness scores

**Figure 9:**
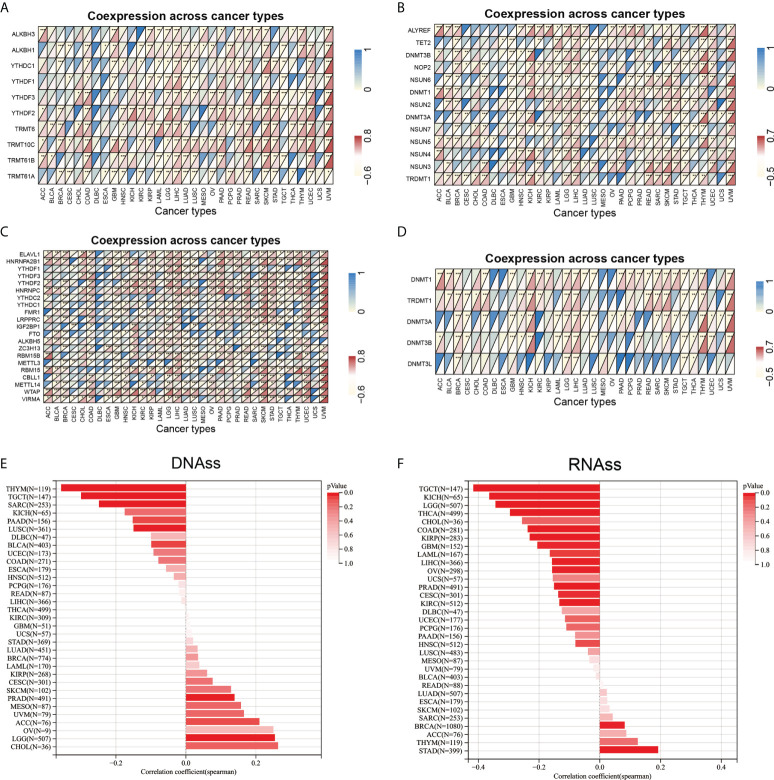
Correlation analysis between ARPC5 expression and RNA modification-related genes, DNA methyltransferases, and tumor stemness score in 33 cancer types. **(A)** Co-expression of ARPC5 with m1A-related genes. **(B)** Co-expression of ARPC5 with m5C-related genes. **(C)** Co-expression of ARPC5 with m6A-related genes. **(D)** Co-expression of ARPC5 with DNA methyltransferases. **(E)** The correlation between ARPC5 expression and Tumor Stemness score (DNAss). **(F)** The correlation between ARPC5 expression and Tumor Stemness score (RNAss). *p < 0.05; **p < 0.01; ***p < 0.001.

The RNA modification had been proved to affect mRNA stability, splicing, and translation and has important oncogenic role or tumor suppressor in different cancer types (26). The association between ARPC5 expression with RNA modification-related genes was showed in **Figures 9A–C**. We found that the expression level of ARPC5 in LGG, LIHC, SKCM, and UVM was significantly related to 10 m1A-related genes, whereas less correlation was observed in ACC, BLCA, CESC, ESCA, KICH, MESO, and SARC; no correlation was detected in DBLC and UCS (**Figure 9A**). Similarly, we examined the co-expression relations between ARPC5 and 13 m5C-related genes expression using Spearman’s coefficient analysis; the results demonstrated that the expression of ARPC5 was associated with most m5C-related genes in LGG, LICH, SKCM, and UVM, whereas the correlation in CHOL, DLBC, ESCA, MESO, and UCS were relatively small (**Figure 9B**). In addition, we found that a great majority of m6A genes in COAD, LGG, LIHC, SKCM, and UVM co-expressed with ARPC5. Less significant connection, even no association between ARPC5 expression and m6A-related genes, was found in CESC, DBLC, ESCA, MESO, and UCS (**Figure 9C**). The above results suggested that ARPC5 may participate in RNA modification and thereby contribute to tumor development in certain cancers.

DNA methylation plays an important regulatory role in the growth, development, gene expression pattern, and genome stability, which is dynamically regulated by DNA methyltransferase and DNA demethylase activities. In our analyses, we discovered that ARPC5 expression was correlated with the expression of four methyltransferases (DNMT1, TRDMT1, DNMT3A, and DNMT3B) in multiple tumors, such as BRCA, LGG, KICH, LIHC, SKCM, THYM, and UVM (**Figure 9D**), whereas DNMT3L was evidently correlated with ARPC5 expression only in five cancer types: BRCA, LGG, LIHC, TGCT, and THCA.

We also analyzed the association of ARPC5 expression with tumor stemness scores (DNAss and RNAss) in 33 cancer types. The results indicated that the ARPC5 was significantly positively correlated with DNAss in PRAD and LGG (*p* < 0.05). In contrast, the expression of ARPC5 in THYM, TGCT, SARS, and LUSC was negatively associated with DNAss (*p* < 0.05) (**Figure 9E**). As for the stem cell score (RNAss), we found the expression level of ARPC5 was prominently positively related to the STAD and BRCA stem cell scores (RNAss) whereas negatively correlated with RNAss in multiple cancer types (*p* < 0.05), such as TGCT, KICH, LGG, THCA, COAD, KIRP, GBM, LAML, LIHC, OV, PRAD, CESC, and KIRC (**Figure 9F**).

### ARPC5 is highly expressed in HCC cells and tissues

**Figure 10:**
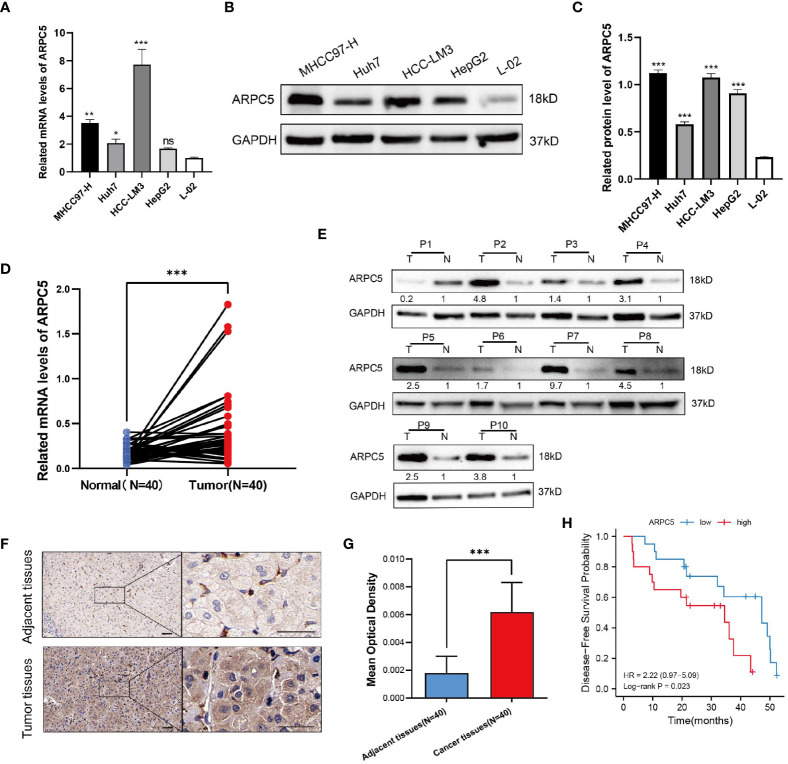
ARPC5 is upregulated in HCC cells and primary HCC tissues. **(A)** qPCR analysis of ARPC5 mRNA expression in four HCC cell lines (MHCC97-H, Huh7, HCC-LM3, and HepG2) and normal liver cell line (LO2). GAPDH was used as an internal control error bars represent *M* ± SEM (triplicate experiments). **(B, C)** The protein expression of ARPC5 was detected in four HCC cell lines and normal liver cell line with Western blot analysis. Error bars represent *M* ± SD of triplicate measurements. **(D)** The mRNA expression of ARPC5 in 40 pairs HCC tissues and adjacent para-carcinoma tissues was evaluated using qPCR. **(E)** Western blot analysis of ARPC5 protein expression in 10 paired HCC tissues and adjacent normal tissues. The number presented the relative protein expression levels of ARPC5. **(F)** Representative images of ARPC5 immunohistochemical staining analysis in the HCC tissue and adjacent normal liver tissue, original magnifications: ×40 and ×200. Scale bars, 50 μm. **(G)** Quantitative analysis of ARPC5 expression in HCC tissues based on mean optical density of immunohistochemical staining. Error bars represent the *M* ± SD of multiple tissues. **(H)** Kaplan–Meier curves showed that higher expression of ARPC5 was associated with poor DFS in HCC patients. *p < 0.05; **p < 0.01; ***p < 0.001. ns, no significance.

In the above expression analysis of bioinformatics method, we found that ARPC5 was significantly upregulated in multiple types of cancer, including LIHC. To further identify the results of bioinformatics analysis, the expression of ARPC5 in HCC cell lines (including MHCC97-H, Huh-7, HCC-LM3, and HepG2) and 40 paired HCC tissues was detected *via* qPCR. The results indicated that the mRNA expression level of ARPC5 was significant higher in three HCC cell lines (MHCC97-H, Huh-7, and HCC-LM3) (**Figure 10A**) and HCC tissues (**Figure 10D**) compared with that in normal liver cell line L-02 and paired adjacent liver tissues, respectively. The results of our experiments were consistent with the results of bioinformatics analysis.

Next, the protein expression level of ARPC5 in HCC cells and HCC tissues were analyzed with Western blot and immunohistochemical staining. We found that the protein expression of ARPC5 in MHCC97-H, Huh-7, HCC-LM3, and HepG2 was significantly higher than in normal live cell line (**Figures 10B, C**). Moreover, ARPC5 was relatively higher in HCC-LM3 and MHCC97-H, which were selected for subsequent functional experiments. Then, 10 of 40 paired HCC tissues were randomly selected for Western blot analysis; the results showed that the ARPC5 expression in most HCC tissues was upregulated compared with the adjacent normal tissues (**Figure 10E**). Furthermore, immunohistochemistry for ARPC5 was conducted in 40 paired HCC samples to verify the results; we found that immunohistochemical staining of ARPC5 was obviously observed in the cytoplasm of HCC cancer tissues, whereas no or weak staining was found in adjacent non-cancerous tissues. The average optical density value of ARPC5 immunohistochemical staining in cancer tissues was higher than adjacent normal liver tissues (**Figures 10F, G**), indicating that the ARPC5 expression was higher in tumor tissues than adjacent normal liver tissues, which cohered with the results of Western blot. Then, the patients were divided into high- and low-expression groups based on the median mRNA expression level of ARPC5, and we further conducted Kaplan–Meier survival analysis to explore the correlation between ARPC5 expression and DFS of HCC patients. The result was showed in **Figure 10H**; we found that high ARPC5 expression was significantly related to a poor DFS in HCC patients (HR = 2.22; 95% CI: 0.97, 5.09; *p* = 0.023). The median DFS of low ARPC5 expression was 47.2 months, and that of high ARPC5 expression group was 34.5 months.

### Downregulation ARPC5 significantly inhibits proliferation and promotes apoptosis of HCC cells

**Figure 11:**
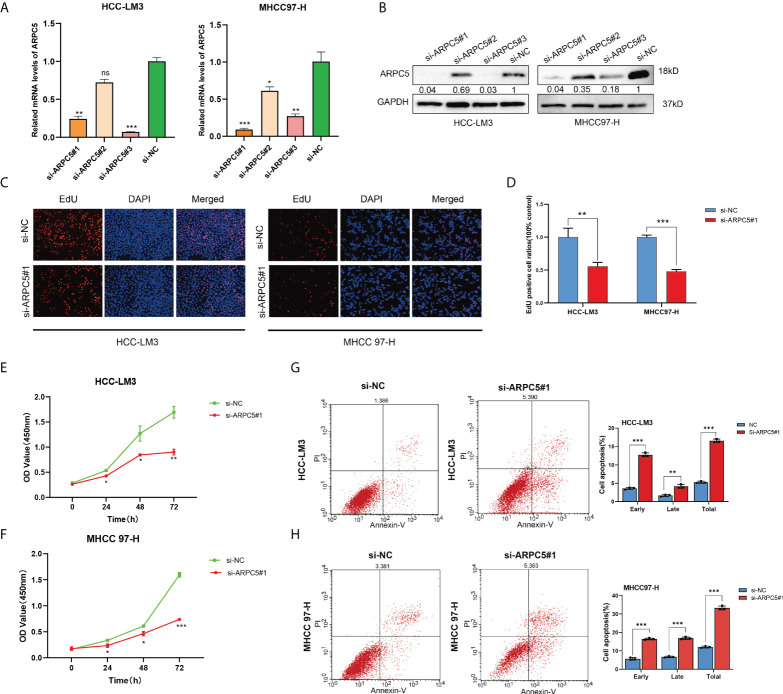
Silencing of ARPC5 inhibits cell proliferation and promotes cell apoptosis of HCC. **(A)** The knockdown efficiency of siRNA–ARPC5 was examined in HCC-LM3 and MHCC97-H cells with qPCR. **(B)** The knockdown efficiency of siRNA-ARPC5 was examined in HCC-LM3 and MHCC97-H cells with Western blot. The number presented as relative protein expression levels of ARPC5. (**C–D**) EdU assays for HCC-LM3 and MHCC 97-H were performed to evaluate cell proliferation ability after transfecting siRNA-ARPC5#1. Representative images **(C)** and the number of proliferative cells were calculated **(D)**; original magnification, ×200. **(E–F)** Cellular growth curves were evaluated by CCK-8 assays in HCC-LM3 and MHCC97-H cells. **(G**–**H)** Flow cytometry was applied to test the apoptosis of HCC cells transfected with si-ARPC5 #1 in HCC-LM3 and MHCC 97-H cells. All data are presented as the *M* ± SD of three independent experiments. *p < 0.05; **p < 0.01; ***p < 0.001. ns, no significance.

In our previous study, we discovered that ARPC5 in HCC mainly participates in MAPK signaling pathway and WNT signaling pathway basing on KEGG enrichment analysis (15). To investigate the potential functions of ARPC5 in HCC cells, we downregulated the expressions of APRC5 and examined the effects of ARPC5 knockdown on cell proliferation and apoptosis. First, qPCR assays and Western blot were performed to verify the transfection efficiency of ARPC5 in both HCC-LM3 and MHCC 97-H cells. The results showed that ARPC5 can be effectively interfered by si-ARPC5#1 and si-ARPC5#3 (**Figures 11A, B**). We selected si-ARPC5#1 for subsequent function experiments. Then, we discussed the effect of ARPC5 on HCC cells proliferation using CCK8 assays and EdU staining assays. The results of EdU assays demonstrated that the silence of ARPC5 distinctly suppressed the proliferative capacity of HCC cells compared with controls (**Figures 11C, D**). The growth curves from CCK-8 assays suggested that proliferation of HCC cells transfected with si-ARPC5 were significantly inhibited compared with that transfected with si-NC (**Figures 11E, F**). Flow cytometry analysis was used to detect the apoptosis of HCC cells transfected with si-ARPC5#1; the results showed the percentage of early and late apoptotic cells significantly increased in HCC-LM3 and MHCC 97-H cells with ARPC5 downregulation (**Figures 11G, H**).

### Knockdown of ARPC5 suppresses the invasion, migration, and epithelial-mesenchymal transition of HCC cells

**Figure 12:**
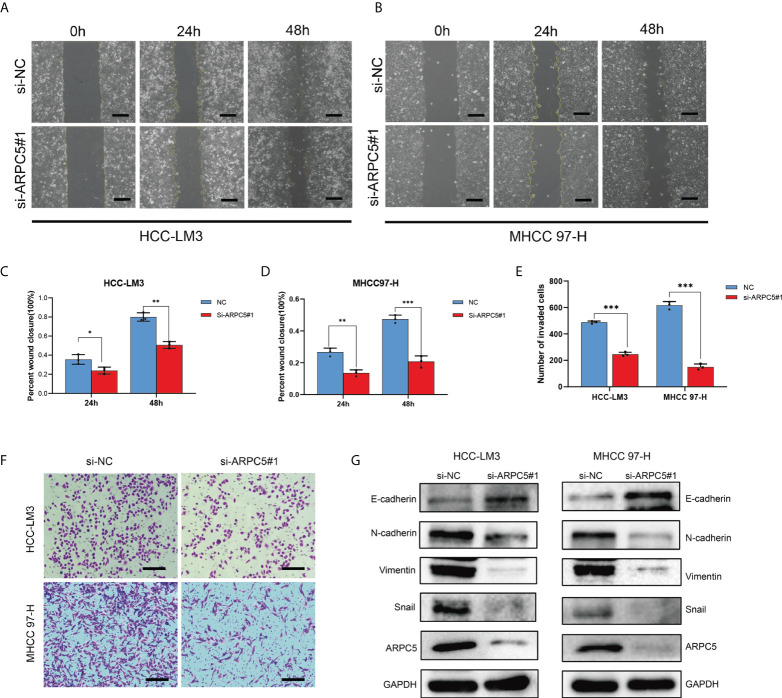
Role of ARPC5 inhibition on migration, invasion, and epithelial–mesenchymal transition (EMT) of HCC cells. **(A–D)** Migration ability was assessed by scratch wound healing assay, representative images **(A, B)** were shown (original magnification, ×200; scale bars, 50 µm), and wound healing areas were calculated **(C, D)**. **(E**, **F)** Transwell assay was applied to examine the invasion ability, representative images **(F)** were shown (original magnification, ×200; scale bars, 50 µm), and the histogram showed the number of invasion cells **(E)**. **(G)** Western blot showed the changes of EMT proteins in HCC-LM3 and MHCC97-H cells transfected with si-ARPC5#1. *p < 0.05; **p < 0.01; ***p < 0.001.

The potential role of APRC5 in the cell migration and invasion was estimated by a scratch wound healing assay and transwell assays. The migration rates of HCC-LM3 and MHCC97-H cells transfected with si-ARPC5 were evidently induced compared with those transfected with si-NC after the scratches were performed for 24 and 48 h (**Figures 12A–D**). Moreover, the number of invasion cells was significantly decreased following ARPC5 knockdown in HCC cells (**Figures 12E, F**). Epithelial-mesenchymal transition (EMT) had been reported as a critical process for tumor invasion and metastasis. We thus further examined the EMT markers (E-cadherin, N-cadherin, vimentin, and snail) by Western blot to investigate whether ARPC5 could affect EMT in HCC cells, the results showed that knockdown of ARPC5 reduced the expression of N-cadherin, Vimentin, and Snail whereas increased E-cadherin expression in HCC-LM3 and MHCC 97-H cells (**Figure 12G**). These results suggested that silencing ARPC5 inhibited HCC cell migration and invasion by suppressing the EMT.

## Discussion

Transcriptomic gene expression analysis offers an optimal opportunity to explore the heterogeneity and complexity of different cancers and to seek new prognostic and therapeutic biomarkers. Several evidence reveals that ARPC5 is associated with tumor progression, metastasis, and prognosis, indicating that ARPC5 may represent a promising biomarker and therapeutic target. Therefore, it is critical to systematically investigate the role of ARPC5 in different types of cancer. In this study, we comprehensively analyzed the expression level of ARPC5 in multiple cancers basing on several different databases. We found that ARPC5 was significantly expressed in 22 tumor tissues compared with corresponding normal tissues and the expression levels was associated with tumor prognosis in multiple cancers. Meanwhile, we also explored the relationship of ARPC5 expression with gene mutation, gene modification, TME, tumor immune infiltration cells, ICIs response, and tumor stemness scores. Notably, we perform a series of experiments to identify the differential expression of ARPC5 in HCC tissues and HCC cells and further revealed that ARPC5 had a cancer-promoting effect in HCC cells and enhanced HCC progression for the first time.

In the present study, we found that ARPC5 expression was significantly upregulated in most cancer types basing on the TCGA datasets integrating with GTEx datasets, including GBM, LGG, BRCA, CESC, ESCA, KIRP, COAD, PRAD, STAD, HNSC, KIRC, LUSC, LIHC, SKCM, BLCA, THCA, OV, PAAD, TGCT, and CHOL. While ARPC5 in UCS and KICH showed lower expression when compared with normal samples. It was reported that the expression of ARPC5 was significantly higher in HNSCC tissues than in non-cancer tissues, and ARPC5 was also significantly increased in invasive cancer cells (13). ARPC5 expression was significantly elevated in tumor tissues of lung squamous cell carcinoma (10). These findings were consistent with our results. To validate the results of bioinformatic analyses, we examined the expression levels of ARPC5 in 40 paired HCC tissues and HCC cell lines. The results manifested the expression of ARPC5 was higher in HCC tissues and HCC cells when compared with correspond adjacent normal liver tissues or normal liver cell both in mRNA and protein levels. The bioinformatic analyses were corroborated by experimental findings.

The prognosis value of ARPC5 was analyzed in 22 cancers in which ARPC5 expression varied significantly between cancer and normal tissues. The survival analysis revealed that ARPC5 was closely associated with survival indicators such as OS, PFS, and DSS. We found that the high expression of ARPC5 was closely linked with poor OS in ESCA, HNSC, KIRC, KIRP, LGG, LIHC, and THCA, with exception for OV and SKCM. Moreover, there were significant negative correlations between ARPC5 expression and PFS in BLCA, HNSC, KIRC, KIRP, LGG, LIHC, and PRAD, whereas positive correlation was observed in LUSC and SKCM. The association of ARPC5 with DSS presented similar results. We found a higher level of ARPC5 expression in KIRC, KIRP, LGG, and LIHC lead to unfavorable prognosis, including OS, PFS, and DSS, whereas ARPC5 in SKCM displayed the opposite results. In addition, we also proved that high expression of ARPC5 was unfavorable for DFS of patients with HCC following curative resection. Previous studies in MM and HCC reported that the high expression of ARPC5 was associated with poor OS and acted as an independent prognostic factor for MM and HCC patients (29, 30). Our current results are in harmony with these previous observations. These results indicating that ARPC5 may functions as an oncogene and represent a new prognostic biomarker for some cancer types. In this study, we found ARPC5 expression was closely correlated with tumor stage, histologic grade, and tumor molecular subtype in pan-cancer analyses. The higher the expression of ARPC5, the more advanced tumor stage for the patients with KIRC and KIRP. The similar results were presented in the correlation between ARPC5 expression and histologic grades in KIRC, LGG, LIHC, and USEC, indicating that ARPC5 can promote tumor progression and facilitate tumor malignancy. Silencing of ARPC5 inhibited cancer cell proliferation in lung squamous cell carcinoma, suggesting that ARPC5 might contribute to lung squamous cell carcinoma development (10). ARPC5 acted as a candidate target of miR-133a in HNSCC, knockdown ARPC5 significant reduced cell migration and invasion of HNSCC cell lines (13). In melanoma, YAP drives ARPC5 expression to enhance cell migration, invasion, and focal adhesions (14). In our study, we conducted a series of functional experiment in HCC cells and discovered downregulation ARPC5 significantly inhibits cell proliferation, migration, invasion, and EMT and promotes cell apoptosis in HCC. To our knowledge, this is the first study focus on the biological functions of ARPC5 in HCC. Nevertheless, the associated mechanisms require further elucidation.

Genetic alternation occurred in the coding region of genes leading to various disease, including tumors. Tumor heterogeneity caused by somatic mutations plays a crucial role in tumor growth and metastasis (31). The frequency of different mutational processes varies among different cancer types. In our analyses, we found that the most frequent mutation type of ARPC5 was “Amplification” mutation and the frequency of “Amplification” was varied among different cancer types, which was most commonly observed in cholangiocarcinoma, invasive breast carcinoma, and HCC. Cancer genomes mutations may be affected by intrinsic DNA replication machinery, mutation exposures, defective DNA repair, and enzymatic modifications of DNA (32). It is widely known that the epigenetic alternation caused by DNA methylation promotes the cancer susceptibility and progression. DNA hypomethylation leads to carcinogenesis and development mainly through transcriptional activation, MSI, and overexpression of oncogenes and loss of imprinting (33, 34). Thus, we further conducted co-expression analysis to explore the correlation between ARPC5 and five DNA methyltransferases (including DNMT1, TRDMT1, DNMT3A, DNMT3B, and DNMT3L). We found that ARPC5 expression was closely correlated with the expression of methyltransferases in most cancer types, especially in KICH and UVM. We speculated that ARPC5 might contribute to cancer progression through influencing the genes stability.

The bidirectional interaction between cancer cells and TME is responsible for tumor development, progression, and drug resistance (35). The TME primarily consists of tumor-infiltrating cells, vasculature, extracellular matrix (ECM), as well as other matrix-associated molecules and have proved to play a significant role in clinical outcomes and response to therapy (36, 37). Immune cells in TME are especially dependent on the proper functioning of the cytoskeletal proteins, for example, Arp2/3 complex (38). Arp2/3 complex plays an essential role in cell migration of T cells, neutrophils and platelets, as well as for CTL assembly (38). The patients with ARPC1B-deficient exhibited a decrease in the number of CD8^+^ T cell and characterized by dysfunctional T cells (39). However, the role of ARPC5 expression in TME, immune cells, and different immune subtypes still remains to be elucidated. Thus, we first explored the correlation of ARPC5 with TME with immune scores and stromal scores calculated by the ESTIMATE algorithms, which can facilitate the quantification of the immune and stromal components in each tumor sample. We found that ARPC5 was evidently positively associated with Immune scores in 22 cancers, and related to Stromal scores in 15 cancer types, suggesting ARPC5 might be a critical driver of immune cells and stromal cells. Then, we performed a more in-depth study to explore which classes of immune cells were associated with ARPC5 expression with TIMER algorithms. The results suggested that the expression of ARPC5 was positively associated with the infiltration level of B cell, CD4^+^ T cell, CD8^+^ T cell, neutrophil cell, macrophage cell, and DC cell in most cancer types, especially for KIRC, LGG, PRAD, THCA, and THYM. In addition, we discovered that ARPC5 was expressed inconsistently in different immune subtypes; ARPC5 was widely highly expressed in C2 subtype and lowly expressed in C3 subtype. These results prompted that ARPC5 have stronger association with certain immune cells involving in IFN-gamma dominant but less relating to inflammatory processes.

In recent years, immune checkpoint inhibitors (ICIs) have revolutionized treatment paradigms and improved survival outcomes of many solid tumors (40). Nevertheless, only a minority of patients can benefit from ICIs with the overall response rates (RRs) no more than 20% (41). In addition, ICIs also come with a unique and sometimes devastating immune-related toxicities. Thus, there is an urgent need to explore biomarkers to accurately predict response and improve treatment selection of ICIs. The PD-L1 expression profiles in cancers have been extensively studied in the past decade. The role of PD-L1 as an effectively predictive biomarker largely based on the results of the KEYNOTE 024 trial study in non-small cell lung cancer, which showed superior outcomes in patients with PD-L1 expression greater than 50% treated with pembrolizumab as the first-line method (42). However, PD-L1 expression was discorded between resected tissues and biopsy specimens, and the expression level varied significantly among different tumor types (43). Currently, effectiveness of PD-L1 detection as an anti-tumor immune response index is still controversial. Recently numerous studies have established the major role of neo-epitopes antigens, resulting from genomic instability status on tumor cells, on cancer immune recognition and specific T-cell activation (44). Tumor with higher TMB, MSI, and neoantigens closely correlates with more T-cell recognition and better clinical outcomes (45, 46). Nevertheless, TMB is independent of PD-L1 status in most cancer types; the combination of TMB, PD-L1, and MSI-H has the better predictive performance of ICIs responsiveness than each alone (41). The present study performed a comprehensive analysis of ARPC5 with existing biomarkers of ICIs including TMB, MSI, neoantigens, and immune checkpoint–related genes in various cancer types. We detected that APRC5 expression was significantly correlated with most of the 47 immune checkpoint–related genes in most cancers, such PRAD, TGCT, KIRC, LIHC, KIRC, THCA, LGG, KICH, and PCPG. In LIHC, ARPC5 was positively related to 39 ICIs-related genes, including PD-1(PDCD1), PD-L1(CD274), PD-L2(PDCD1LG2), and CTLA4. Moreover, ARPC5 had a correlation with TMB in 12 cancers, MSI in 12 cancers, and neoantigens in five cancers. Interestingly, ARPC5 expression in STAD and BRCA was positively associated with TMB, MSI, and neoantigens. These findings suggested that the ARPC5 can be used as a new biomarker to predict ICIs response for certain cancers.

In summary, comprehensive analyses were conducted in our study to explore the expression patterns and prognostic values of ARPC5 in pan-cancer using multiple databases. We discovered that the expression of ARPC5 was upregulated in most cancer types and high-expressed ARPC5 was associated with poor survival outcomes and tumor progression in some cancers. In addition, we found that ARPC5 was closely related to TME, tumor infiltration immune cells, immune subtypes, and biomarkers of ICIs, which might provide a new insight of ARPC5 with tumor immunity and would be favorable for mining novel therapeutic target and predictive biomarker for immunotherapy. Moreover, this study was the first to validate the differential expression of ARPC5 in HCC tissues and explore the role of ARPC5 in the proliferation, apoptosis, and invasion of HCC cells, which provided a preliminary foundation for the development of biomarker-targeting therapies in HCC.

## Data availability statement

The original contributions presented in the study are included in the article/**Supplementary Material**. Further inquiries can be directed to the corresponding author.

## Ethics statement

The studies involving human participants were reviewed and approved by The Second Affiliated Hospital of Nanchang University Medical Research Ethics Committee. The patients/participants provided their written informed consent to participate in this study.

## Author contributions

JW and SH contributed to conception and design of the study. SH, LS, and PH organized the database. SH and KL performed the statistical analysis. SH wrote the first draft of the manuscript. LS, PH, and KL wrote sections of the manuscript. All authors contributed to manuscript revision, read, and approved the submitted version.

## Funding

This research was supported by the National Natural Science Foundation of China (NO. 82060435).

## Conflict of interest

The authors declare that the research was conducted in the absence of any commercial or financial relationships that could be construed as a potential conflict of interest.

## Publisher’s note

All claims expressed in this article are solely those of the authors and do not necessarily represent those of their affiliated organizations, or those of the publisher, the editors and the reviewers. Any product that may be evaluated in this article, or claim that may be made by its manufacturer, is not guaranteed or endorsed by the publisher.

## Glossary

**Table:**

ARPC5	actin-related protein 2/3 complex subunit 5
Arp2/3	actin-related protein 2/3
TCGA	the Cancer Genome Atlas database
GTEx	Genotype-Tissue Expression database
TIMER	Tumor Immune Evaluation Resource
FPKM	Fragments Per Kilobase per Million
CNV	copy number variations
TMB	tumor mutation burden
MSI	microsatellite instability;
MMRs	mismatch repairs
HCC	hepatocellular carcinoma
OS	overall survival
DSS	disease-specific survival
PFI	progression-free interval
DFS	disease-free survival
TME	tumor microenvironment
ICIs	immune checkpoint inhibitors
m6A	N6methyladenosine
m5C	RNA5methylcytosine
m1A	N1methyladenosine
qPCR	quantitative real-time polymerase chain reaction
siRNA	small-interfering RNA
EdU	5-ethynyl-2′-deoxyuridine
ACC	adrenocortical carcinoma
BLCA	bladder urothelial carcinoma
BRCA	breast invasive carcinoma
CESC	cervical squamous cell carcinoma and endocervical adenocarcinoma
CHOL	cholangiocarcinoma
COAD	colon adenocarcinoma
DLBC	lymphoid neoplasm diffuse large B cell lymphoma
ESCA	esophageal carcinoma
GBM	glioblastoma multiforme
HNSC	head and neck squamous cell carcinoma
KICH	kidney chromophobe
KIRC	kidney renal clear cell carcinoma
KIRP	kidney renal papillary cell carcinoma
LAML	acute myeloid leukemia;
LGG	brain lower grade glioma
LIHC	liver hepatocellular carcinoma;
LUAD	lung adenocarcinoma
LUSC	lung squamous cell carcinoma;
MESO	mesothelioma
OV	ovarian serous cystadenocarcinoma
PAAD	pancreatic adenocarcinoma
PCPG	pheochromocytoma and paraganglioma
PRAD	prostate adenocarcinoma
READ	rectum adenocarcinoma
SARC	sarcoma
SKCM	skin cutaneous melanoma;
STAD	stomach adenocarcinoma
TGCT	testicular germ cell tumors;
THCA	thyroid carcinoma
THYM	Thymoma
UCEC	uterine corpus endometrial carcinoma
UCS	uterine carcinosarcoma
UVM	uveal melanoma
