# Corrigendum: A comprehensively prognostic and immunological analysis of actin-related protein 2/3 complex subunit 5 in pan-cancer and identification in hepatocellular carcinoma

**DOI:** 10.3389/fimmu.2022.1047151

**Published:** 2022-10-17

**Authors:** Shenglan Huang, Liying Sun, Ping Hou, Kan Liu, Jianbing Wu

**Affiliations:** ^1^ Department of Oncology, The Second Affiliated Hospital of Nanchang University, Nanchang, China; ^2^ Jiangxi Key Laboratory of Clinical and Translational Cancer Research, The Second Affiliated Hospital of Nanchang University, Nanchang, China; ^3^ Department of Hepatobiliary Surgery, The Second Affiliated Hospital of Nanchang University, Nanchang, China

**Keywords:** ARPC5, prognosis, biomarker, immune, pan-cancer, hepatocellular carcinoma

In the published article, there was an error in **Figures 10B, C** as published. The ARPC5 and GAPDH was not in the same running gel even though the electrophoresis was performed in the same buffer with the consistent protein samples, but the GAPDH in **Figure 10B** was not the internal reference of ARPC5. The corrected **Figure 10** and its caption “**Figure 10** ARPC5 is upregulated in HCC cells and primary HCC tissues. **(A)** qPCR analysis of ARPC5 mRNA expression in four HCC cell lines (MHCC97-H, Huh7, HCC-LM3, and HepG2) and normal liver cell line (LO2). GAPDH was used as an internal control error bars represent *M* ± SEM (triplicate experiments). **(B, C)** The protein expression of ARPC5 was detected in four HCC cell lines and normal liver cell line with Western blot analysis. Error bars represent *M* ± *SD* of triplicate measurements. **(D)** The mRNA expression of ARPC5 in 40 pairs HCC tissues and adjacent para-carcinoma tissues was evaluated using qPCR. **(E)** Western blot analysis of ARPC5 protein expression in 10 paired HCC tissues and adjacent normal tissues. The number presented the relative protein expression levels of ARPC5. **(F)** Representative images of ARPC5 immunohistochemical staining analysis in the HCC tissue and adjacent normal liver tissue, original magnifications: ×40 and ×200. Scale bars, 50 μm. **(G)** Quantitative analysis of ARPC5 expression in HCC tissues based on mean optical density of immunohistochemical staining. Error bars represent the *M* ± *SD* of multiple tissues. **(H)** Kaplan–Meier curves showed that higher expression of ARPC5 was associated with poor DFS in HCC patients. *p < 0.05; **p < 0.01; ***p < 0.001. ns, no significance.” appear below.

**Figure 10:**
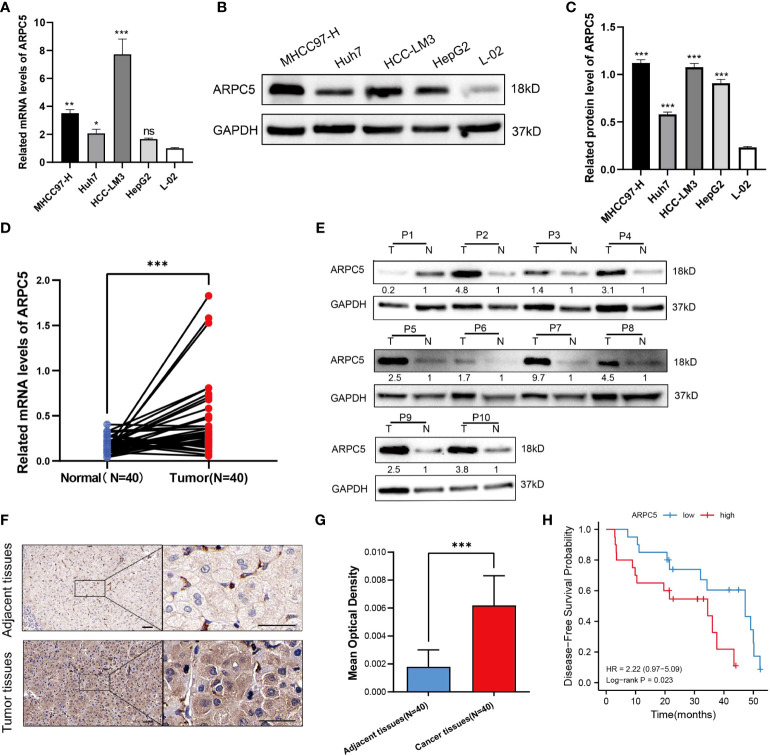
ARPC5 is upregulated in HCC cells and primary HCC tissues. **(A)** qPCR analysis of ARPC5 mRNA expression in four HCC cell lines (MHCC97-H, Huh7, HCC-LM3, and HepG2) and normal liver cell line (LO2). GAPDH was used as an internal control error bars represent M ± SEM (triplicate experiments). **(B, C)** The protein expression of ARPC5 was detected in four HCC cell lines and normal liver cell line with Western blot analysis. Error bars represent M ± SD of triplicate measurements. **(D)** The mRNA expression of ARPC5 in 40 pairs HCC tissues and adjacent para-carcinoma tissues was evaluated using qPCR. **(E)** Western blot analysis of ARPC5 protein expression in 10 paired HCC tissues and adjacent normal tissues. The number presented the relative protein expression levels of ARPC5. **(F)** Representative images of ARPC5 immunohistochemical staining analysis in the HCC tissue and adjacent normal liver tissue, original magnifications: ×40 and ×200. Scale bars, 50 μm. **(G)** Quantitative analysis of ARPC5 expression in HCC tissues based on mean optical density of immunohistochemical staining. Error bars represent the M ± SD of multiple tissues. **(H)** Kaplan–Meier curves showed that higher expression of ARPC5 was associated with poor DFS in HCC patients. *p < 0.05; **p < 0.01; ***p < 0.001. ns, no significance.

The authors apologize for this error and state that this does not change the scientific conclusions of the article in any way. The original article has been updated.

## Publisher’s note

All claims expressed in this article are solely those of the authors and do not necessarily represent those of their affiliated organizations, or those of the publisher, the editors and the reviewers. Any product that may be evaluated in this article, or claim that may be made by its manufacturer, is not guaranteed or endorsed by the publisher.

