# Supplementary figures and images for: A comprehensively prognostic and immunological analysis of actin-related protein 2/3 complex subunit 5 in pan-cancer and identification in hepatocellular carcinoma
Source: Front Immunol. 2022 Sep 6;13:944898. doi: 10.3389/fimmu.2022.944898 (PMC9485570; doi:10.3389/fimmu.2022.944898)

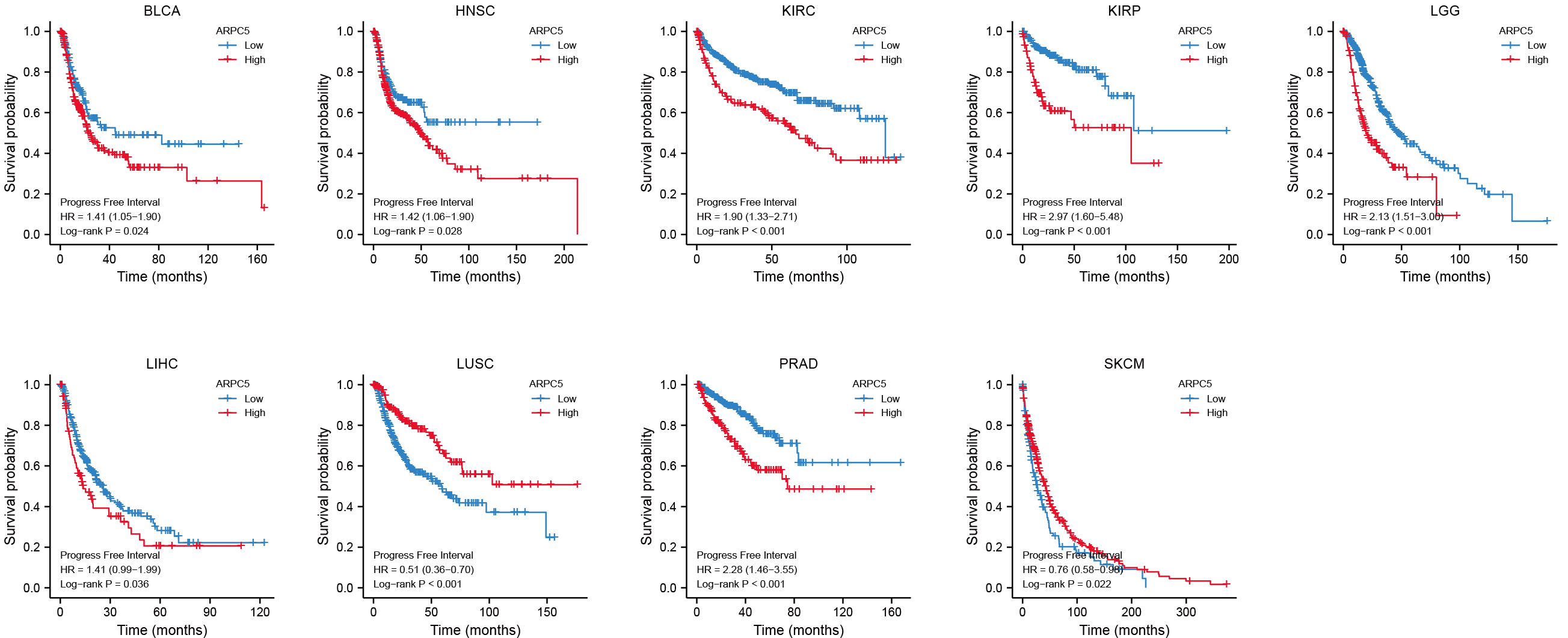

Supplement: Supplementary file 1 [file Image_1.tif]

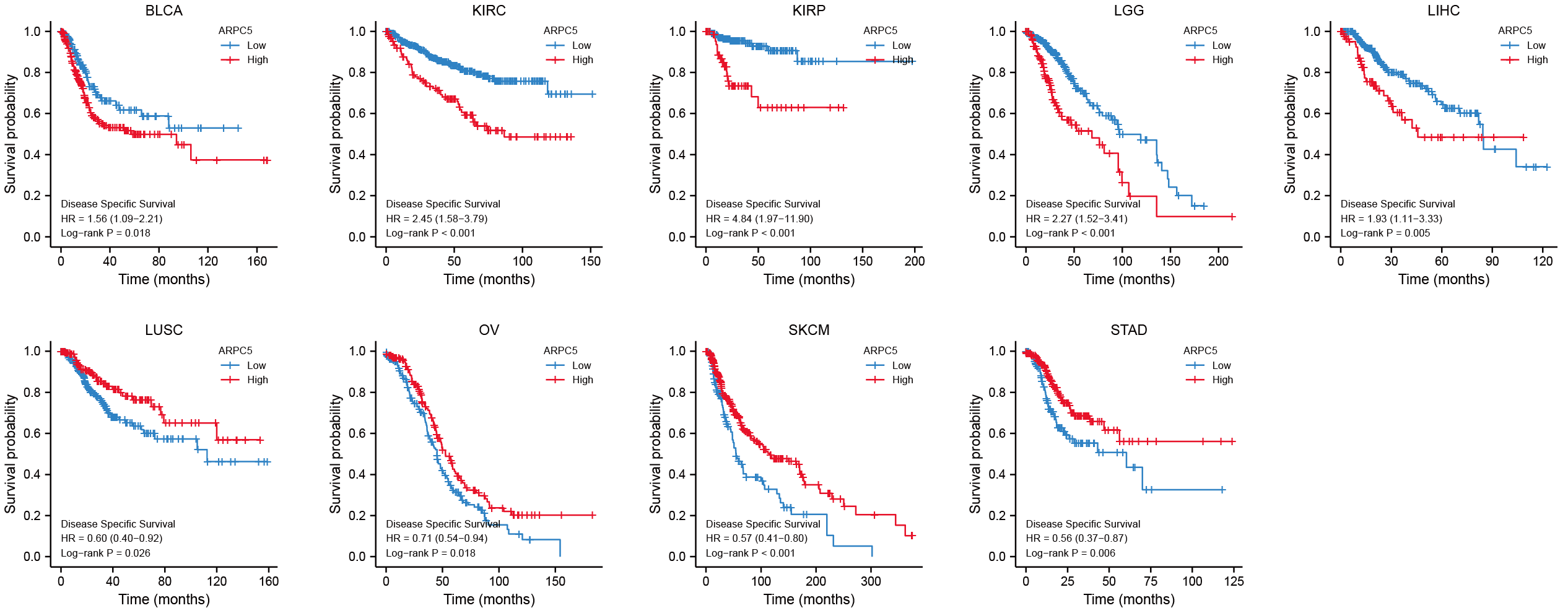

Supplement: Supplementary file 2 [file Image_2.tif]
